# Modeling the ratio of correlated biomarkers using copula regression

**DOI:** 10.1177/09622802241313293

**Published:** 2025-02-11

**Authors:** Moritz Berger, Nadja Klein, Michael Wagner, Matthias Schmid

**Affiliations:** 1 Department of Medical Biometry, Informatics and Epidemiology, Faculty of Medicine, University of Bonn, Bonn, Germany; 2 Scientific Computing Center, 150232 Karlsruhe Institute of Technology, Karlsruhe, Baden-Württemberg, Germany; 3 German Center for Neurodegenerative Diseases Bonn, Nordrhein-Westfalen, Germany

**Keywords:** Copula model, distributional regression, gamma distribution, negative dependence, ratio outcome

## Abstract

Modeling the ratio of two dependent components as a function of covariates is a frequently pursued objective in observational research. Despite the high relevance of this topic in medical studies, where biomarker ratios are often used as surrogate endpoints for specific diseases, existing models are commonly based on oversimplified assumptions, assuming e.g. independence or strictly positive associations between the components. In this paper, we overcome such limitations and propose a regression model where the marginal distributions of the two components are linked by a copula. A key feature of our model is that it allows for both positive and negative associations between the components, with one of the model parameters being directly interpretable in terms of Kendall’s rank correlation coefficient. We study our method theoretically, evaluate finite sample properties in a simulation study and demonstrate its efficacy in an application to diagnosis of Alzheimer’s disease via ratios of amyloid-beta and total tau protein biomarkers.

## 1. Introduction

A common objective in medical research is to analyze the ratio of two (possibly dependent) components $U,V\in R^{+}$ .^1^ Typical examples are, among others, (i) the low-density lipoprotein (LDL)/high-density lipoprotein (HDL) cholesterol ratio in cardiovascular research,^2^ defined as the ratio of the LDL and the HDL concentrations in plasma or serum, (ii) the CD4/CD8 ratio in HIV research,^3^ which measures the ratio of T helper cells to cytotoxic T cells in the human immune system, (iii) the testosterone over epitestosterone (T/E) ratio in antidoping research,^4^ and (iv) the GEFC/REFC ratio in ophthalmic research, corresponding to the green and red emission components in fundus autofluorescence imaging.^5^ In many of such studies, biomarker ratios are used as early indicators or even as surrogate endpoints for a specific disease. In these cases, the focus is not only on the characterization of the marginal ratio distribution, but also on modeling this distribution as a function of a set of covariates $X=(X_{1},\ldots ,X_{p}{)}^{⊤}$ .^6^

When setting up a model relating the ratio outcome R=U/V to the covariates X , a common assumption is that both components follow either log-normal or gamma distributions, thereby accounting for the nonnegativity of the component values and the skewness of their distributions.^7,8^ In the former case it is easily derived that the ratio is again log-normally distributed. The latter case, which will be dealt with in this paper, is considerably less straightforward but is often preferred in practice due to its increased efficiency.^9–12^

In the special case where U and V are *independently* gamma distributed, the ratio R=U/V follows a generalized beta distribution of the second kind, in the following abbreviated by *GB2*.^13^ A regression approach for the GB2 distribution has been proposed by Tulupyev et al.,^14^ who studied the determinants of alcohol abuse in HIV-positive persons using the framework of vector generalized additive models.^15^ Other recent contributions that employ the GB2 distribution include.^16–19^

The case of *correlated* gamma distributed components has earlier been studied by Lee, Holland and Flueck^20^ and Tubbs.^21^ Based on Kibble’s bivariate gamma distribution for (U,V) , Berger et al^12^ developed the *extended GB2 (eGB2) model* for the ratio of two *positively correlated* gamma distributed components. Their model is characterized by three parameters, of which one is directly interpretable in terms of the Pearson correlation coefficient between the two components. Conceptually, the extended GB2 model can be seen as a distributional regression model embedded in the framework of generalized additive models for location, scale and shape (GAMLSS).^6,22^

Despite its major importance in medical studies, no regression modeling strategy exists (to the best of our knowledge) for ratio outcomes with two *negatively correlated* gamma distributed components. Negatively correlated measurements are encountered in numerous applications, for example in dementia research, where ratios of cerebrospinal fluid (CSF) biomarkers are used for the early diagnosis of Alzheimer’s disease (AD).^23^ Importantly, measurements of the widely employed amyloid- β 42 protein and total tau protein biomarkers are known to exhibit a negative correlation.^24^ In recent publications, the Gaussian regression model has been used for modeling ratios of CSF biomarkers (e.g., Xu et al.).^25^ Clearly, this model neither accounts for the characteristics of the bivariate distribution of (U,V) nor for the skewness in the distribution of the ratio outcome R .

Motivated by these problems, and to address the current shortcomings in modeling ratio outcomes with negatively correlated components, we propose a regression model where the joint bivariate distribution of the two gamma distributed components is defined by a copula.^26^ Depending on the specific copula, the model can flexibly account for either negative or positive associations between the two components (measured by Spearman’s or Kendall’s rank correlation coefficient). It also allows for modeling different characteristics of the two marginal distributions, including possibly unequal rate and shape parameters. By relating the covariates X to the parameters of the marginals, as well as to the association parameter defined by the copula, our model further allows to derive the conditional probability density function (PDF) of R|X as a function of covariates. This, in turn, allows for the analysis of conditional distributional parameters (like the expected value, median or quantiles), including valid inferential conclusions for these quantities.

Compared to earlier work of Berger et al^12^ and Berger and Schmid,^6^ the approach presented in this paper has a number of advantages: First, it allows for both positive *and* negative correlations between the components U and V , whereas the previous approaches are restricted to positive correlations. It therefore applies to a much broader range of medical applications, including the analysis of amyloid- β 42 protein and total tau protein biomarkers in dementia research (see above). Second, although our main focus is on gamma distributed marginals, our new model allows for a large number of alternative marginal distributions (in particular, the log-normal distribution). Consequently, our model is much more flexible in approximating the distributional shapes of observed biomarker values in medical studies, even allowing for mixed types of marginals (e.g. gamma and log-normal or even non-continuous markers). Third, although we showcase our model using the Frank copula in Section 2, the proposed approach extends to many other copula models. This allows for modeling a variety of dependence structures, as we elaborate further in Section 5. We emphasize that, in contrast to the proposed approach, Berger et al^12^ and Berger and Schmid^6^ required very specific forms of bivariate distributions, e.g. Kibble’s bivariate gamma distribution or the bivariate power-normal distribution.

We apply the new approach to data from a multi-center observational cohort study conducted by the German Dementia Competence Network (DCN).^27^ Study participants were diagnosed with either mild cognitive impairment (MCI), AD, or other dementia. The study aims at determining the diagnostic and prognostic power of clinical, laboratory and imaging methods. This task is considered to be a major challenge, as the period from the first clinical symptoms of AD to disease onset might take years to decades.^28^ Consequently, as biomarker ratios like the amyloid- β 42/total tau ratio are considered to be strong predictors of AD progression, it is of high interest to relate these measurements to patient characteristics like age, sex and educational level.^29^ As will be demonstrated in Section 4, the proposed copula regression model can be suitably applied to address this problem, resulting in meaningful descriptive and inferential findings regarding the associations between the biomarker ratio and individual patient characteristics.

The rest of the paper is organized as follows: Section 2 derives the distributional copula regression model, states theoretical results with implications for the interpretation of covariate effects, and presents estimation, prediction and inference. The efficacy of our approach is demonstrated empirically in a simulation study in Section 3 and in our main application to AD progression in Section 4. The main findings of the paper are discussed in Section 5.

## 2. Methods

Motivated by the needs of our application, in Section 2.1 we derive the distribution of the ratio of two gamma distributed components with dependence induced by the Frank copula.^30,31^ Details on model specification and fitting are given in Section 2.2. Section 2.3 covers the prediction of distributional parameters and inference.

### 2.1. Distributional concept

Let U and V be two gamma distributed random variables with PDFs

(1) $$f_{U}(u)=\frac{\lambda_{U}^{\delta_{U}}}{\Gamma (\delta_{U})}u^{\delta_{U}-1}\exp\;(-\lambda_{U}u)\;\;\;\text{and}\;\;\;f_{V}(v)=\frac{\lambda_{V}^{\delta_{V}}}{\Gamma (\delta_{V})}v^{\delta_{V}-1}\exp\;(-\lambda_{V}v)$$

where $\lambda_{U}$ , $\lambda_{V}$ >0 denote the rate parameters and $\delta_{U}$ , $\delta_{V}$ >0 denote the shape parameters of $f_{U}$ and $f_{V}$ , respectively. To allow for positive and negative dependencies between U and V , we model their joint distribution using the Frank copula with copula function $C_{\theta }$ . By Sklar’s theorem, the joint distribution of (U,V) is thus given by

(2) $$\begin{array}{rl} F_{U,V}(u,v) & =C_{\theta }\left(F_{U}(u),F_{V}(v)\right)\\ & =-\frac{1}{\theta }\;\log\;\left\{1+\frac{[\exp\;(-\theta F_{U}(u))-1][\exp\;(-\theta F_{V}(v))-1]}{\exp\;(-\theta )-1}\right\} \end{array}$$

where $F_{U,V}$ , $F_{U}$ and $F_{V}$ denote the joint bivariate and marginal cumulative distribution functions (CDFs) of U and V , respectively.^31^ The parameter θ∈R∖{0} determines the association between U and V . It can be shown that Kendall’s rank correlation coefficient τ is a monotone increasing function of θ , given by

(3) $$\tau (\theta )=1\;+\;\frac{4}{\theta }\left(\frac{1}{\theta }\int_{0}^{\theta }\frac{t}{e^{t}-1}\;dt-1\right)$$

that can take any value in [−1,1] .^30,32^ As a consequence, the CDF in (2) allows for (possibly highly) positive or negative correlations between the two components U and V . The joint PDF of (U,V) is given by

(4) $$\begin{array}{rl} f_{U,V}(u,v) & =\frac{\partial^{2}}{\partial u\partial v}F_{U,V}(u,v)=c_{\theta }(F_{U}(u),F_{V}(v))\;f_{U}(u)\;f_{V}(v)\\ & =\frac{-\theta \;\exp\;(-\theta F_{U}(u))\;\exp\;(-\theta F_{V}(v))\;(\exp\;(-\theta )-1)\;f_{U}(u)\;f_{V}(v)}{{\left\{(\exp\;(-\theta )-1)+[\exp\;(-\theta F_{U}(u))-1]\;[\exp\;(-\theta F_{V}(v))-1]\right\}}^{2}} \end{array}$$

where $c_{\theta }(a,b):=\partial^{2}/(\partial a\partial b)\;C_{\theta }(a,b)$ is the PDF of the Frank copula.

Remark 1 In general our method also works for other parametric copulas $C_{\theta }$ and marginal distributions $F_{U}$ , $F_{V}$ , respectively. We still chose to showcase and detail or distributional concept for the special case of the Frank copula with gamma marginals because fitting this model to the joint distribution of amyloid- β 42 and total tau yielded the best fit in terms of BIC compared to Gaussian, Clayton, Joe and Gumbel copulas in our application.

We derive the resulting PDF of the ratio R=U/V , an interpretable representation thereof and the CDF in the following three propositions.

Proposition 1 Let the PDF of (U,V) be defined by (4). Then the PDF of the ratio R:=U/V (with r>0 ) is given by

(5) $$\begin{array}{rl} f_{R}(r)=\int_{0}^{1} & \left|F_{V}^{-1}(s)\right|\;c_{\theta }\left[F_{U}(r\;F_{V}^{-1}(s)),s\right]\;f_{U}(rF_{V}^{-1}(s))\;ds\\ =\int_{0}^{1} & \frac{\exp\;\left[-\theta \;F_{U}(r\;F_{V}^{-1}(s))\right]\;\exp\;(-\theta s)\;(-\theta )\;(\exp\;(-\theta )-1)}{{\left\{(\exp\;(-\theta )-1)+[\exp\;[-\theta \;F_{U}(r\;F_{V}^{-1}(s))]-1][\exp\;(-\theta s)-1]\right\}}^{2}}\\ & \times F_{V}^{-1}(s)\;f_{U}(r\;F_{V}^{-1}(s))\;ds \end{array}$$

where |⋅| denotes the absolute value function.

Proof. Proposition 1 is derived from Proposition 1 of Ly et al.,^33^ who provided analytical results for the PDF of the quotient U/V of two random variables whose dependence structure can be described by an absolutely continuous copula.□

**Figure 1.**
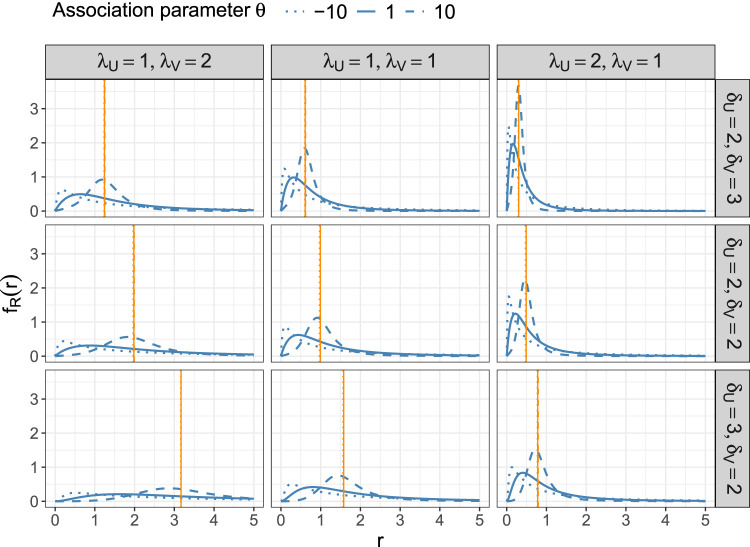
Examples of the PDF of R=U/V derived in Proposition 1 for parameters $\lambda_{U},\lambda_{V}\in \{1,2\}$ , $\delta_{U},\delta_{V}\in \{2,3\}$ and θ∈{−10,1,10} (corresponding to rank correlation coefficients τ∈{−0.67,0.11,0.67} ). In each panel the three lines refer to θ=−10 (dotted), θ=1 (solid) and θ=10 (dashed). Vertical, orange lines refer to the median values of R .

Remark 2 Figure 1 visualizes the PDF of R for different values of the rate, shape and association parameters. The figure illustrates that the form of the PDF is strongly related to the ratio of marginal means $E(U)/E(V)=\lambda_{V}\delta_{U}/\lambda_{U}\delta_{V}$ , which is highest in the lower left panel ( E(U)/E(V)=3 ) where the dispersion is very large, and lowest in the upper right panel ( E(U)/E(V)=1/3 ) where the PDFs are heavily right-skewed. Figure 1 also describes the association between the PDF and the Kendall’s rank correlation coefficient. In each of the nine cases the mode of the PDF increases as θ increases. Last, our illustration may indicate that the median of R does not vary with θ (as it is equal for the three PDFs in each panel). We do not give a formal proof here, but the finding is meaningful as the same holds for the ratio of two log-normally distributed components, where the median depends on the mean parameter, only.

Proposition 2 Let the PDF of (U,V) be defined by (4). Then the PDF of the random variable R in (5) can be re-written as

(6) $$\begin{array}{rl} f_{R}(r) & =\int_{0}^{1}c_{\theta }\left\{\frac{1}{\Gamma (\delta_{U})}\;\gamma \left(\delta_{U},r\;\Lambda \;\gamma^{-1}\left(\delta_{V},\Gamma (\delta_{V})s\right)\right),s\right\}\\ & \;\times \;\frac{\Lambda^{\delta_{U}}\;r^{\left(\delta_{U}-1\right)}}{\Gamma (\delta_{U})}{\left[\gamma^{-1}\left(\delta_{V},\Gamma (\delta_{V})s\right)\right]}^{\delta_{U}}\\ & \;\times \;\exp\;\left[-r\;\Lambda \;\gamma^{-1}\left(\delta_{V},\Gamma (\delta_{V})s\right)\right]\;ds \end{array}$$

where $\Lambda :=\lambda_{U}/\lambda_{V}$ denotes the ratio of the two rate parameters and γ(⋅,⋅) is the lower incomplete gamma function.

Proof. The proof of Proposition 2 is given in Appendix A.

**Figure 2.**
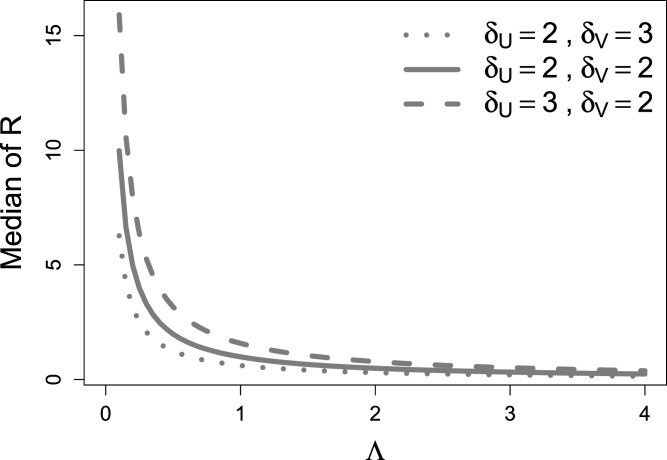
Median of R=U/V for parameters Λ∈[0.1,4] , $\delta_{U},\delta_{V}\in \{2,3\}$ and θ=1 fixed, as calculated from the formula in Proposition 3. The parameter on the x-axis corresponds to the ratio of the two rate parameters $\Lambda =\lambda_{U}/\lambda_{V}$ .

Remark 3 By Proposition 2 the PDF of R can be written as a function of the ratio of the rate parameters $\Lambda =\lambda_{U}/\lambda_{V}$ . This facilitates the interpretation of the proposed regression model introduced in the next Section 2.2. Figure 2 illustrates how the median of R is related to Λ . It suggests that the median decreases monotonically in Λ .

Proposition 3 Let the CDF of (U,V) be defined by (2). Then the CDF of the random variable R is given by

(7) $$F_{R}(r)=\int_{0}^{1}\;\frac{A\;f_{U}(r\;F_{V}^{-1}(s))\;\frac{r}{f_{V}(F_{V}^{-1}(s))}\;(\exp\;(-\theta s)-1)+(A-1)\exp\;(-\theta s)}{\exp\;(-\theta )+(A-1)\exp\;(-\theta s)-A}\;ds$$

where $A=\exp\;[-\theta \;F_{U}(rF_{V}^{-1}(s))]$ .

Proof. By equation (9) of Ly et al.,^33^ the CDF of R is derived as

$$\begin{array}{rl} F_{R}(r) & =\underset{=0}{\underbrace{F_{V}(0)}}+\int_{0}^{1}\underset{=1}{\underbrace{\text{sgn}(F_{V}^{-1}(s))}}\;\frac{\partial }{\partial s}C_{\theta }\left[F_{U}(r\;F_{V}^{-1}(s)),s\right]\;ds\\ & =-\frac{1}{\theta }\;\int_{0}^{1}\frac{\partial }{\partial s}\log\;\left\{1+\frac{[\exp\;[-\theta \;F_{U}(rF_{V}^{-1}(s))]-1]\;[\exp\;(-\theta s)-1]}{\exp\;(-\theta )-1}\right\}\;ds\\ & =\int_{0}^{1}\{\frac{\exp\;[-\theta \;F_{U}(rF_{V}^{-1}(s))]\;f_{U}(r\;F_{V}^{-1}(s))\;\frac{r}{f_{V}(F_{V}^{-1}(s))}\;(\exp\;(-\theta s)-1)}{\exp\;(-\theta )-1+[\exp\;[-\theta \;F_{U}(rF_{V}^{-1}(s))]-1]\;[\exp\;(-\theta s)-1]}\\ & \;+\frac{[\exp\;[-\theta \;F_{U}(rF_{V}^{-1}(s))]-1]\;\exp\;(-\theta s)}{\exp\;(-\theta )-1+[\exp\;[-\theta \;F_{U}(rF_{V}^{-1}(s))]-1]\;[\exp\;(-\theta s)-1]}\}ds \end{array}$$

where sgn(⋅) is the sign function. Rearrangement of the last equation gives (7).□

### 2.2. Regression specification and estimation

To model the entire distribution of R as a function of covariates $X=(X_{1},\ldots ,X_{p}{)}^{⊤}$ , we propose to relate both the logarithmic rate parameters $\lambda_{U}$ and $\lambda_{V}$ and the association parameter θ to predictors of the form

(8) $$\begin{array}{rl} \log\;(\lambda_{U}|X) & =\eta_{U}=\beta_{U0}+\beta_{U1}X_{1}+\ldots +\beta_{Up}X_{p} \end{array}$$

(9) $$\begin{array}{rl} \log\;(\lambda_{V}|X) & =\eta_{V}=\beta_{V0}+\beta_{V1}X_{1}+\ldots +\beta_{Vp}X_{p}\;\text{and} \end{array}$$

(10) $$\begin{array}{rl} \theta |X & =\eta_{\theta }=\beta_{\theta 0}+\beta_{\theta 1}X_{1}+\ldots +\beta_{\theta p}X_{p} \end{array}$$

where $\beta_{U}=(\beta_{U0},\ldots ,\beta_{Up}{)}^{⊤}$ , $\beta_{V}=(\beta_{V0},\ldots ,\beta_{Vp}{)}^{⊤}$ and $\beta_{\theta }=(\beta_{\theta 0},\ldots ,\beta_{\theta p}{)}^{⊤}$ are sets of real-valued coefficients. Analogous to classical gamma regression (Chapter 5.3 of Fahrmeir et al.,^34^) the use of the logarithmic transformation in (8) and (9) ensures positivity of the rate parameters. Since θ∈R∖{0} , no transformation is needed for the association parameter. As a result of (8) and (9) it holds that $\log\;(\Lambda |X)=\eta_{U}-\eta_{V}$ . Based on this representation, covariate effects with regard to the rate parameters can be investigated using one-dimensional hypothesis tests and p -values, see also our application in Section 4.

Remark 4 In principle, our approach allows to make use of the full flexibility of GAMLSS by relating all distributional parameters (including the shape parameters $\delta_{U},\delta_{V}$ ) to the covariates and by including nonlinear effects in the predictors. However, in our application we found that the specification in (8) to (10) provides a sufficient fit, thereby meeting a compromise between model fit and model complexity. Furthermore, it greatly simplifies the interpretation of the results (as we will further elaborate in Section 4). Based on these considerations, we assume that the shape parameters $\delta_{U}$ and $\delta_{V}$ do not depend on X , but can be treated as nuisance parameters.

Definition 1 In the following, we denote the regression model for the ratio R=U/V with the distribution from Proposition 3 and with covariate-dependent parameters as specified in (8) to (10) by the Frank copula with gamma distributed marginals (FCGAMs). The FCGAM model imposes the constraint $\delta_{U}$ , $\delta_{V}$ >1 to ensure that the two marginals both exhibit a unimodal, right-skewed distribution, which is the common form of biomarker distributions in medical applications.

Corollary 1 For a set of i.i.d. observations $(u_{1},v_{1},x_{1}^{⊤}{)}^{⊤},\ldots ,(u_{n},v_{n},x_{n}^{⊤}{)}^{⊤}$ with ratios $r_{1}=u_{1}/v_{1},\ldots ,r_{n}=u_{n}/v_{n}$ and model coefficients $\gamma =(\beta_{U}^{⊤},\beta_{V}^{⊤},\beta_{\theta }^{⊤},\delta_{U},\delta_{V}{)}^{⊤}$ , the log-likelihood function of the FCGAM model is given by

(11) $$\begin{array}{rl} & \ell (\beta_{U},\beta_{V},\beta_{\theta },\delta_{U},\delta_{V};u_{1},\ldots ,u_{n},v_{1},\ldots ,v_{n},x_{1},\ldots ,x_{n})\\ & \;=\sum_{i=1}^{n}\;\{\log\;[f_{U,V}(u_{i},v_{i}|x_{i},\beta_{U},\beta_{V},\beta_{\theta },\delta_{U},\delta_{V})]\}\\ & \;=\sum_{i=1}^{n}\;\{\log\;[\exp\;(-x_{i}^{⊤}\beta_{\theta }\;F_{U}(u_{i};x_{i}^{⊤}\beta_{U},\delta_{U}))\exp\;(-x_{i}^{⊤}\beta_{\theta }\;F_{V}(v_{i};x_{i}^{⊤}\beta_{V},\delta_{V}))\\ & \;\times \;(-x_{i}^{⊤}\beta_{\theta })(\exp\;(-x_{i}^{⊤}\beta_{\theta })-1)f_{U}(u_{i};x_{i}^{⊤}\beta_{U},\delta_{U})\;f_{V}(v_{i};x_{i}^{⊤}\beta_{V},\delta_{V})]\\ & \;-2\log\;[(\exp\;(-x_{i}^{⊤}\beta_{\theta })-1)+(\exp\;(-x_{i}^{⊤}\beta_{\theta }\;F_{U}(u_{i};x_{i}^{⊤}\beta_{U},\delta_{U}))-1)\\ & \;\times \;(\exp\;(-x_{i}^{⊤}\beta_{\theta }\;F_{V}(v_{i};x_{i}^{⊤}\beta_{V},\delta_{V}))-1)]\} \end{array}$$

Corollary 2 Under the usual regularity assumptions, the estimator

(12) $$\begin{array}{rl} \hat{\gamma } & =({\hat{\beta }}_{U}^{⊤},{\hat{\beta }}_{V}^{⊤},{\hat{\beta }}_{\theta }^{⊤},{\hat{\delta }}_{U},{\hat{\delta }}_{V}{)}^{⊤}\\ & :=\underset{\gamma =\beta_{U},\beta_{V},\beta_{\theta },\delta_{U},\delta_{V}}{\mathrm{argmax}}\ell (\beta_{U},\beta_{V},\beta_{\theta },\delta_{U},\delta_{V};u_{1},\ldots ,u_{n},v_{1},\ldots ,v_{n},x_{1},\ldots ,x_{n}) \end{array}$$

is consistent and asymptotically normal for n→∞ .

While Corollary 2 shows asymptotic normality when n→∞ , for finite samples, reliable confidence intervals can be obtained via a Bayesian approach, see Section 2.3 below for details.

#### 2.2.1. Implementational details

Maximization of the log-likelihood function in (11) can be carried out using the R function FCGAMoptim(), which is part of the supplemental material to this paper. The optimization algorithm is based on the Broyden-Fletcher-Goldfarb-Shanno algorithm implemented in the R function optim(), setting the additional constraint $\delta_{U}$ , $\delta_{V}$ >1 .

### 2.3. Prediction of distributional parameters and inference

#### 2.3.1. Prediction

For a new observation with covariate values $\tilde{x}$ , predictions of the conditional PDF $f_{R}(r|\tilde{x})$ can be obtained by computing the maximum likelihood estimate (MLE) and by plugging the estimated parameters $\hat{\Lambda }=\exp\;({\tilde{x}}^{⊤}{\hat{\beta }}_{U}-{\tilde{x}}^{⊤}{\hat{\beta }}_{V})$ , $\hat{\theta }={\tilde{x}}^{⊤}{\hat{\beta }}_{\theta }$ and ${\hat{\delta }}_{U}$ , ${\hat{\delta }}_{V}$ in equation (6). The predicted PDF can then be used to predict any distributional parameter of interest (like the expected value, median or quantiles). For example, denoting the predicted PDF by ${\hat{f}}_{R}(r|\tilde{x})$ , the predicted median can be calculated by

(13) $${\hat{r}}_{\text{med}}\;|\;\tilde{x}=\text{min}\{r\in R^{+}\;|\;\int_{0}^{r}{\hat{f}}_{R}(s|\tilde{x})\;ds\geq 0.5\}$$

#### 2.3.2. Inference

Despite the asymptotic results from Corollary 2, more reliable finite-sample confidence intervals have been established in additive models.^35^ This is particularly the case for the quantities of interest here (such as the median of R above). The reason is that these are nonlinear transformations of the original model coefficients such that confidence intervals would show an additional finite-sample bias due to the application of the Δ -rule. Following Wood,^35^ we thus propose to construct confidence intervals of γ using a Bayesian approach, which we accordingly refer to as credible intervals. Assuming flat priors p(γ)∝const on γ , the posterior distribution of γ is approximated by

(14) $$\gamma \;|\;u_{1},\ldots ,u_{n},v_{1},\ldots ,v_{n}\;∼\;N(\hat{\gamma },J^{-1}(\hat{\gamma }))$$

where $J(\hat{\gamma })$ is the Hessian of the negative log-likelihood evaluated at $\hat{\gamma }$ (equation (6.26) of Wood^35^). Consequently, approximate (1−α)% credible intervals for the coefficients γ can be obtained by drawing a large sample from the posterior distribution (14) and by calculating the α/2 and (1−α/2) percentiles from this sample Wood.^35^^(p.293)^ In our simulations (Section 3) and in the analysis of the DCN study data (Section 4) we used samples of size 10,000 throughout.

## 3. Simulations

We conducted three simulation studies to investigate the performance of the FCGAM model. Our main aims were (a) to analyze the model fit and the coverage of the credible intervals, (b) to evaluate how the performance of the FCGAM approach is affected by the sample size and the choice of the association parameter θ , and (c) to benchmark our method against alternative ones, in particular against the extended GB2 model by Berger et al^12^ which assumes the correlation between U and V to be positive.

### 3.1. Experimental design

In all simulations the ratio outcome was generated according to the PDF of the FCGAM model derived in Proposition 1. Similar to the application data in Section 4, we considered two standard normally distributed covariates $X_{1},X_{2}∼N(0,1)$ and two binary covariates $X_{3},X_{4}∼B(1,0.5)$ , which were pairwise equi-correlated with Pearson correlation coefficient 0.4, resulting in the covariance matrix

(15) $$\begin{array}{r} \Sigma =\left(\begin{array}{cccc} 1 & 0.4 & 0.4 & 0.4\\ 0.4 & 1 & 0.4 & 0.4\\ 0.4 & 0.4 & 1 & 0.4\\ 0.4 & 0.4 & 0.4 & 1 \end{array}\right) \end{array}$$

For each n∈{200,500,1000} we simulated 1000 independent data sets.

In *Simulation Study 1*, we considered scenarios with fixed *negative* correlation (the case which motivated our development of the FCGAM model), setting $\beta_{\theta 0}\in \{-1,-5,-10\}$ and $\beta_{\theta 1}=\ldots =\beta_{\theta 4}=0$ . This resulted in the respective rank correlation coefficients τ∈{−0.11,−0.46,−0.67} . The rate parameters were related to the four covariates through the coefficients $\beta_{U}=(0,0.4,-0.4,0.2,-0.2{)}^{⊤}$ and $\beta_{V}=(0,-0.2,0.2,-0.4,0.4{)}^{⊤}$ . The shape parameters were set to $\delta_{U}=2$ and $\delta_{V}=6$ in all scenarios.

In *Simulation Study 2*, we considered scenarios with fixed *positive* correlation (the case which has already been covered by the eGB2 model but also applies to the FCGAM model), setting $\beta_{\theta 0}\in \{1,5,10\}$ and $\beta_{\theta 1}=\ldots =\beta_{\theta 4}=0$ . This resulted in the respective rank correlation coefficients τ∈{0.11,0.46,0.67} . To ensure that the outcome values were in a meaningful range (comparable to *Simulation Study 1*) we set the regression coefficients to $\beta_{U}=(0,0.4,-0.4,0.2,-0.2{)}^{⊤}$ and $\beta_{V}=(0,0.2,-0.2,0.4,-0.4{)}^{⊤}$ , and the shape parameters to $\delta_{U}=2$ and $\delta_{V}=2$ .

In *Simulation Study 3*, we evaluated how the model fit of the FCGAM model was affected when falsely assuming a dependence of θ on $X_{1},\ldots ,X_{4}$ , or when ignoring a present dependence of θ on $X_{1},\ldots ,X_{4}$ . For this we reconsidered the data sets from *Simulation Study 1* with τ=−0.11 , the data sets from *Simulation Study 2* with τ=0.11 , and additionally considered scenarios where the association parameter θ was related to the four covariates through the coefficient vector $\beta_{\theta }=(0,1,-1,0.5,-0.5{)}^{⊤}$ (resulting in covariate-dependent rank correlation coefficients $\tau_{i}$ , with the remaining parameters as in *Simulation Study 1*). In all of the three cases we fitted the FCGAM model with covariate-dependent θ (according to (10)) and with constant $\theta =\beta_{\theta 0}$ .

#### 3.1.1. Benchmark methods

We evaluated the fits of the 1000 FCGAM models by computing the predictive log-likelihood values on 1000 independent test data sets. The test data sets (of size n each) were also used to compare the FCGAM model to alternative models. To this purpose, we evaluated the predictive log-likelihood values of the following benchmark methods, where (ii), (iii) and (vi) are univariate regression models for R , (iv) is a univariate regression model for log(R) , and (v) and (vii) are distributional regression models:

(i) The copula-based *FCGAM* model introduced in Section 2.2.
(ii) The extended GB2 model (*eGB2*^12^) assuming a positive correlation between U and V .
(iii) The GB2 model (*GB2*) assuming zero Pearson correlation between U and V .
(iv) A Gaussian regression model with log-transformed ratio outcome values (*LN*).
(v) A Gaussian GAMLSS with log-transformed ratio outcome values, where both the mean and the standard deviation were related to the covariates (*LN.LSS*). The standard deviation was modeled using the log link.
(vi) A Gamma regression model with the original ratio outcome values (*GA*). The mean parameter was related to the covariates and was modeled using the log link.
(vii) A Gamma GAMLSS with the original ratio outcome values, where both the mean and the scale parameters were related to the covariates (*GA.LSS*) using the log link.

### 3.2. Results

#### 3.2.1. Point estimates of the FCGAM coefficients

**Figure 3.**
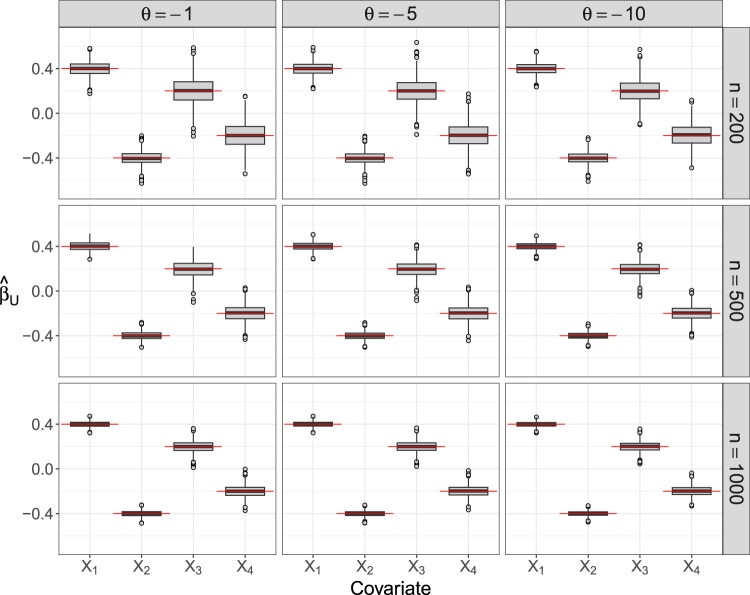
Point estimates of the FCGAM coefficients in *Simulation Study 1*. The boxplots visualize the MLEs of the coefficients $\beta_{U1}=0.4$ , $\beta_{U2}=-0.4$ , $\beta_{U3}=0.2$ and $\beta_{U4}-0.2$ that were obtained from fitting the FCGAM model to 1000 data sets of size n each. The red lines refer to the true values of the coefficients. FCGAM: Frank copula with Gamma Distributed Marginal; MLE: maximum likelihood estimate.

Figure 3 presents the coefficient estimates ${\hat{\beta }}_{U}$ in *Simulation Study 1* with negative (but covariate-independent) correlation between U and V . The boxplots show that on average the estimated coefficients are very close to the true ones, regardless of the association parameter θ . Accordingly, the finite-sample bias of the MLEs is small in all scenarios (with varying n and θ ). From Figure 3 it can also be seen that, as expected, the variance of the estimates decreases with increasing sample size, in particular for the two binary covariates $X_{3}$ and $X_{4}$ . In contrast, the correlation (determined by the value of θ ) has only a small impact on the variance of the estimates. The coefficient estimates ${\hat{\beta }}_{V}$ (presented in Supplemental Figure S1) exhibit even smaller variances in the scenarios with n=500 and n=1000 .

The coefficient estimates ${\hat{\beta }}_{U}$ and ${\hat{\beta }}_{V}$ from *Simulation Study 2* with positive correlation between U and V are shown in Supplemental Figures S2 and S3, respectively. In both cases the bias is small throughout all scenarios. Regarding the variance of the estimates, the results are largely the same as in Figure 3.

#### 3.2.2. Coverages of credible intervals

The coverages of 95% credible intervals obtained from the FCGAM fits are presented in Table 1. They range between 0.928 and 0.958 (*Simulation Study 1*) and between 0.928 and 0.962 (*Simulation Study 2*), which is close to the nominal coverage of 95%. There were only minor differences with regard to sample size and the correlation coefficient. This result demonstrates that not only point estimation but also inference works well for highly positive or negative correlations and fairly small samples.

**Table 1.** Coverage proportions of the Frank copula with Gamma distributed marginal (FCGAM) credible intervals.

Simulation Study 1		$\beta_{U1}$	$\beta_{U2}$	$\beta_{U3}$	$\beta_{U4}$	$\beta_{V1}$	$\beta_{V2}$	$\beta_{V3}$	$\beta_{V4}$
n=200	θ=−1	0.938	0.932	0.936	0.938	0.949	0.949	0.954	0.950
	θ=−5	0.958	0.942	0.955	0.941	0.943	0.935	0.946	0.952
	θ=−10	0.937	0.938	0.932	0.947	0.952	0.946	0.940	0.949
n=500	θ=−1	0.949	0.937	0.940	0.938	0.952	0.953	0.946	0.930
	θ=−5	0.954	0.938	0.928	0.935	0.952	0.932	0.935	0.949
	θ=−10	0.934	0.948	0.939	0.951	0.955	0.947	0.958	0.943
n=1000	θ=−1	0.949	0.937	0.955	0.947	0.944	0.946	0.948	0.937
	θ=−5	0.947	0.942	0.936	0.936	0.957	0.950	0.950	0.950
	θ=−10	0.933	0.945	0.934	0.953	0.943	0.948	0.949	0.943
Simulation Study 2		$\beta_{U1}$	$\beta_{U2}$	$\beta_{U3}$	$\beta_{U4}$	$\beta_{V1}$	$\beta_{V2}$	$\beta_{V3}$	$\beta_{V4}$
n=200	θ=1	0.935	0.929	0.934	0.935	0.947	0.933	0.959	0.954
	θ=5	0.952	0.940	0.954	0.941	0.949	0.951	0.954	0.947
	θ=10	0.938	0.939	0.939	0.947	0.951	0.944	0.948	0.953
n=500	θ=1	0.941	0.948	0.936	0.928	0.940	0.942	0.946	0.940
	θ=5	0.948	0.941	0.929	0.929	0.952	0.944	0.941	0.962
	θ=10	0.931	0.947	0.937	0.951	0.948	0.944	0.949	0.937
n=1000	θ=1	0.950	0.944	0.954	0.944	0.952	0.939	0.953	0.935
	θ=5	0.955	0.942	0.934	0.934	0.956	0.952	0.949	0.947
	θ=10	0.928	0.946	0.934	0.955	0.935	0.956	0.948	0.945

For each coefficient $\beta_{Uj}$ , j=1,…,4, and $\beta_{Vj}$ , j=1,…,4, the table contains the coverage proportion of the 95% credible interval, as obtained from fitting the FCGAM model to 1000 independent data sets of size n each.

#### 3.2.3. Distributional prediction

The root mean squared error (RMSE) of the estimated conditional median values computed from (13) are given in Table 2. In * Simulation Study 1* the performance is quite similar for all three values of θ , whereas in *Simulation Study 2* the RMSE considerably decreases with increasing value of θ . This indicates that estimating the median value works best for highly positive correlations where the PDF of R is rather diffuse with a large mode value (compare Figure 1). It is also seen from Table 2 that the means and the standard deviations of the RMSE decrease with increasing sample size.

**Table 2.** RMSE of the estimated conditional median values of R .

Simulation Study 1		θ=−1	θ=−5	θ=−10
n=200		0.076 (0.035)	0.084 (0.039)	0.081 (0.037)
n=500		0.049 (0.021)	0.053 (0.023)	0.051 (0.021)
n=1000		0.034 (0.015)	0.038 (0.016)	0.036 (0.016)
Simulation Study 2		θ=1	θ=5	θ=10
n=200		0.154 (0.056)	0.101 (0.035)	0.063 (0.021)
n=500		0.097 (0.033)	0.064 (0.022)	0.040 (0.014)
n=1000		0.069 (0.023)	0.046 (0.015)	0.029 (0.010)

Notes: The table presents the mean RMSE of the estimated conditional median of R , as obtained from fitting the FCGAM model to 1000 independent data sets of size n each. Standard deviations of the RMSE values (across the 1000 data sets) are given in brackets. FCGAM: Frank copula with Gamma distributed marginal; RMSE: root mean squared error.

#### 3.2.4. Comparison to alternative models

**Figure 4.**
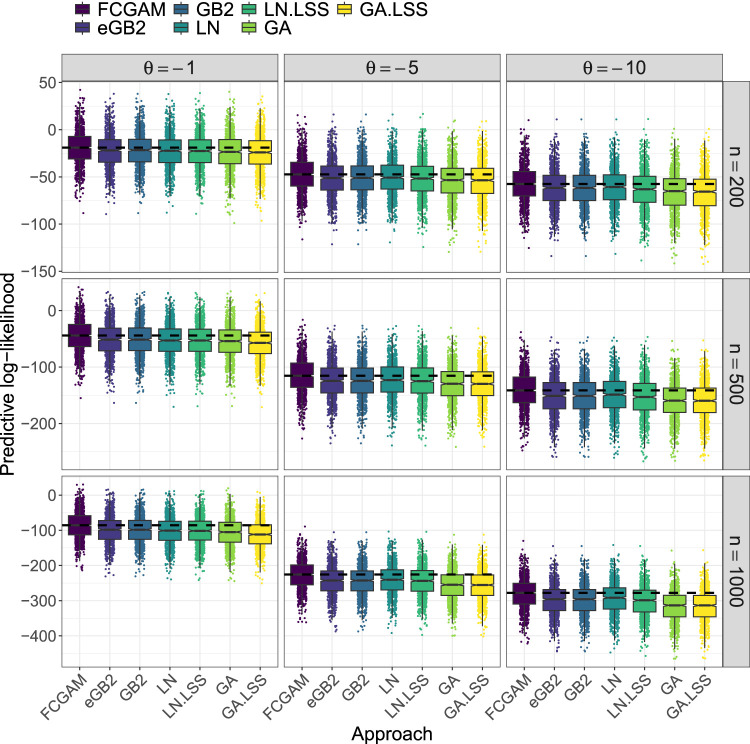
Comparison of the FCGAM: Frank copula with Gamma distributed marginal (FCGAM) model to alternative methods in *Simulation Study 1*. The boxplots visualize the predictive log-likelihood values obtained from the FCGAM model and from the benchmark methods (ii) to (vii). All models were fitted to 1000 independent data sets and evaluated on independently generated test data sets of the same size. In each panel, the dashed horizontal line indicates the median predictive log-likelihood of the best performing method.

Figure 4 and Supplemental Figure S4 show the prediction accuracy (i.e. the predicted log-likelihood values on the test sets) of the FCGAM model and the benchmark methods (ii) to (vi). In *Simulation Study 1* with negative correlation, it can be observed that the FCGAM model achieves the highest accuracy in all scenarios (Figure 4). This difference in accuracy is even more evident when the sample size and the value of the correlation coefficient are increased. The extended GB2 and simple GB2 methods yield similar performances as the Gaussian models with log-transformed outcome (LN and LN.LSS), whereas the Gamma regression models (GA and GA.LSS) result in the lowest accuracy. For both LN and GA the GAMLSS models are not superior to their simple counterparts.

In *Simulation Study 2* with positive correlation, the results change considerably (Supplemental Figure S4). As expected, the performance of the FCGAM and eGB2 models is largely the same, as the eGB2 model also assumes gamma distributed components with positive correlation. The simple GB2 model (assuming uncorrelated components) and the Gaussian models with log-transformed outcomes (LN and LN.LSS) perform comparably well in the scenarios with θ=1 , but deteriorated with increasing correlation ( θ=5 and θ=10 ). Again, the Gamma regression models (GA and GA.LSS) exhibit the worst performance.

#### 3.2.5. Misspecified models for the association parameter in Simulation Study 3

The RMSE of the estimated conditional median values and the predictive log-likelihood values of the FCGAM fits are summarized in Table 3. It is seen that ignoring the dependence of θ on the covariates (in scenarios with covariate-dependent correlation) decreases both the predictive ability and the model fit. In the scenario with n=1000 (large sample size), the difference in predictive log-likelihood values of 5.093 suggests “considerably less” empirical support for the model with constant θ (according to the rules of thumb provided in Burnham and Anderson^36^). On the other hand, when unnecessarily modeling the dependence of θ on $X_{1},\ldots ,X_{4}$ (in scenarios with fixed negative or fixed positive correlation) the predictive ability and the model fit are mostly unaffected (showing only negligible differences in the RMSE and the predictive log-likelihood values).

**Table 3.** Analysis of misspecified models for the association parameter in simulation study 3.

RMSE of median		Modeled θ	Constant θ
Covariate-dependent correlation	n=200	0.069 (0.031)	0.082 (0.042)
	n=500	0.042 (0.018)	0.058 (0.026)
	n=1000	0.031 (0.013)	0.049 (0.021)
Fixed negative correlation	n=200	0.079 (0.032)	0.077 (0.034)
	n=500	0.050 (0.020)	0.047 (0.018)
	n=1000	0.036 (0.015)	0.034 (0.014)
Fixed positive correlation	n=200	0.157 (0.050)	0.152 (0.056)
	n=500	0.103 (0.032)	0.097 (0.033)
	n=1000	0.072 (0.023)	0.069 (0.023)
Predictive log-likelihood		Modeled θ	Constant θ
Covariate-dependent correlation	n=200	−6.769 (19.176)	−7.116 (19.133)
	n=500	−10.736 (29.031)	−12.853 (29.087)
	n=1000	−18.237 (41.177)	−23.330 (40.965)
Fixed negative correlation	n=200	−20.165 (19.163)	−19.356 (19.056)
	n=500	−44.585 (29.092)	−43.979 (29.050)
	n=1000	−85.656 (40.989)	−85.071 (40.967)
Fixed positive correlation	n=200	−295.086 (19.382)	−293.729 (19.324)
	n=500	−732.918 (31.618)	−731.777 (31.509)
	n=1000	−1459.324 (43.363)	−1458.163 (43.359)

The table presents the mean RMSE of the estimated conditional median values (upper part) and the mean of the predictive log-likelihood values (lower part), as obtained from fitting the FCGAM model to 1000 independent data sets and evaluating the fits on 1000 independently generated test data sets. Standard deviations (across the 1000 data sets) are given in brackets. We compared the results of scenarios with covariate-dependent correlation coefficients (in the observed range $\tau_{i}\in [-0.483,\ldots ,0.464]$ ), scenarios with fixed negative correlation τ=−0.11 and scenarios with fixed positive correlation τ=0.11 . The terms “modeled θ ” and “constant θ ” refer to the FCGAM models with a covariate-dependent predictor function for θ (as in (10)) and an intercept-only predictor function for θ ( $\beta_{\theta 1}=\ldots =\beta_{\theta p}=0$ in (10)), respectively. FCGAM: Frank copula with Gamma distributed marginal; RMSE: root mean squared error.

#### 3.2.6. Overall summary

Taken together, we make the following key empirical observations:

1. Point estimates from the FCGAM model are reliable and nearly unbiased even for small sample sizes.
2. The FCGAM model outperforms the eGB2 model in case of negative correlation and is en par with the eGB2 model when the correlation is positive.
3. Falsely modeling the association parameter does not deteriorate predictive performance to a large degree, whereas the FCGAM model with covariate-dependent θ improves the fit when the true association depends on the covariates.

## 4. Cohort study of the german dementia competence network

### 4.1. Background

The multi-center cohort study conducted by the German DCN^27^ enrolled patients aged older than 50 years that were diagnosed with either MCI, ADor other dementia. Recruitment took place between 2003 and 2007. The main objective of the original study was to establish biomarkers for the diagnosis and prognosis of AD using clinical, laboratory and imaging measurements. Here, we investigate covariates that are potentially associated with amyloid- β 42, amyloid- β 40 and total tau protein concentrations measured in CSF samples. These analyses are of high relevance for clinical routine in the neurosciences, since biomarkers enable the detection of AD pathology long before the occurrence of the first clinically obvious symptoms.^28^ Thus, relating covariates to biomarker values provides insight into disease pathology and prevention at the individual patient level. In the neurosciences, amyloid- β 42, amyloid- β 40 and total tau protein concentrations are usually not analyzed separately but in terms of their ratios. More specifically, the amyloid- β 42/40 ratio and amyloid- β 42/total tau ratio are considered to be strong predictors of AD progression.^23^ Therefore we focus on the group of MCI patients and relate their ratios to patient-related risk factors for dementia.

### 4.2. Description of the data

**Figure 5.**
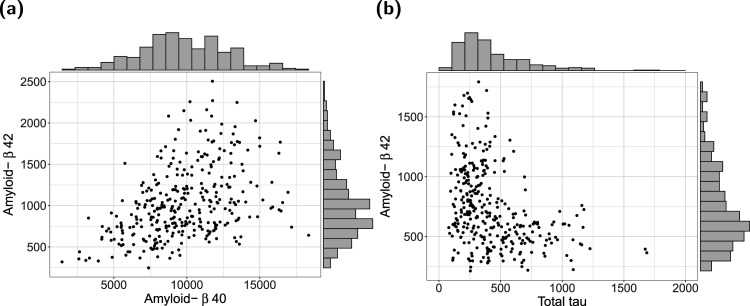
Analysis of the DCN study data. Marginal distributions and joint distributions of (a) amyloid- β 42 and amyloid- β 40, and (b) amyloid- β 42 and total tau in patients with MCI ( n=330 ). Note: Following standard procedures, the study investigators used different PET scans to compute the amyloid- β 42/40 and amyloid- β 42/total tau ratios. For this reason, the distributions of amyloid- β 42 differ between panels (a) and (b). Also note that we excluded an extreme outlier with a total tau >5000 (pg/ml) in panel (b). DCN: Dementia Competence Network; MCI: mild cognitive impairment.

In the DCN study, amyloid- β and total tau baseline concentrations were measured in 374 patients diagnosed with MCI. In all other MCI patients, CSF biosamples were not collected due to either logistic reasons or lack of consent to the invasive procedure of lumbar puncture. Exclusion of patients that did not meet the eligibility criterion (age ≤ 50 years; 7 patients) and of patients with missing values in at least one of the considered risk factors ( 37 patients) resulted in an analysis data set of n=330 patients. For details on the handling of missing values we refer to Berger et al.^12^ The marginal distributions of the components and the joint distributions of (amyloid- β 42, amyloid- β 40) and (amyloid- β 42, total tau) are visualized in Figure 5. The unconditional Kendall’s rank correlation coefficient between the two components is given by τ=0.307 (amyloid- β 42/40) and τ=−0.269 (amyloid- β 42/total tau). This observation suggests the need for a model that can handle both positive and negative correlations between the ratio components and was a major motivation for the development of the FCGAM model, as mentioned before. The unconditional distributions of the amyloid- β 42/40 ratio and the amyloid- β 42/total tau ratio are visualized in Supplemental Figure S5. While the values of the amyloid- β 42/40 ratios are all smaller than 0.3, the amyloid- β 42/total tau ratios range between 0.2 and 13, exhibiting a heavily right-skewed distribution.

The risk factors included in the analysis are summarized in Table 4. These were: (i) sex, (ii) age in years, (iii) educational level (measured by the number of years of education), and (iv) a binary variable indicating whether a patient was a carrier of the apolipoprotein E ϵ 4 (ApoE ϵ 4) allele, which is a strong genetic predictor of AD.

**Table 4.** Description and summary statistics of the two ratio outcomes and the covariates used for the analysis of the DCN study data ( Q1=first quartile,Q3=third quartile ).

Variable	Summary statistics						
	min	Q1	Median	Q3	max	Mean	sd
Amyloid- β 42/40	0.03	0.08	0.10	0.14	0.26	0.11	0.04
Amyloid- β 42/total tau	0.19	0.91	2.13	3.72	12.95	2.70	2.34
Age (years)	51	60	66	73	89	66.51	8.11
Education (years)	2	11	11	13	19	12.18	2.96
Sex	male:	194 (58.8%)			female:	136 (41.2%)	
ApoE ϵ 4	no:	182 (55.2%)			yes:	148 (44.8%)	

All numbers refer to a subset of patients diagnosed with MCI ( n=330 ). For details on the collection of the data, see Kornhuber et al. DCN: Dementia Competence Network; MCI: mild cognitive impairment.^27^

### 4.3. Model fitting I

In a preliminary analysis, we fitted GA and GA.LSS models for the components amyloid- β 42, amyloid- β 40 and total tau, where either the rate parameters only (GA) or both the rate and the shape parameters (GA.LSS) were related to the four covariates. According to the Bayesian information criterion (BIC) the simple GA models (BIC =4899.988 for amyloid- β 42, BIC =6248.201 for amyloid- β 40 and BIC =4574.974 for total tau) showed better fits than the respective GA.LSS models (BIC =4914.396 for amyloid- β 42, BIC =6266.375 for amyloid- β 40 and BIC =4581.902 for total tau). This result indicates that it is sufficient to relate the two rate parameters to the covariates. Furthermore, it supports the assumptions of the proposed FCGAM model, which treats the shape parameters $\delta_{U}$ and $\delta_{V}$ as nuisance parameters.

The fits of the FCGAM model with covariate-dependent association parameter are presented in Supplemental Table S1. According to the credible intervals given in columns 4 and 6, none of the risk factors is found to affect the association parameter θ . Applying equation (3) yielded the mean estimated rank correlations $\hat{\tau }(\hat{\theta })=0.35$ (range: 0.21–0.49) for amyloid- β 42/40 and $\hat{\tau }(\hat{\theta })=-0.23$ (range: −0.41 to 0.03) for amyloid- β 42/total tau. Both estimates are close to the respective unconditional rank correlations.

### 4.4. Model fitting II

Based on the above findings and to further reduce model complexity, we fitted FCGAM models with constant association parameter θ (setting the coefficients $\beta_{\theta ,\text{Age}},\ldots ,\beta_{\theta ,\text{ApoE }\epsilon 4}$ to zero). We then calculated the BIC from these reduced models along with their counterparts obtained from the models with covariate-dependent θ . For amyloid- β 42/40, the BIC values were 11,063.74 (constant θ ) and 11,084.75 (modeled θ ). For amyloid- β 42/total tau, the BIC values were 9249.99 (constant θ ) and 9267.485 (modeled θ ). This result suggests that the reduced models with constant θ meet a better compromise between model fit and model complexity than the respective full models with covariate-dependent θ .

### 4.5. Main results

**Figure 6.**
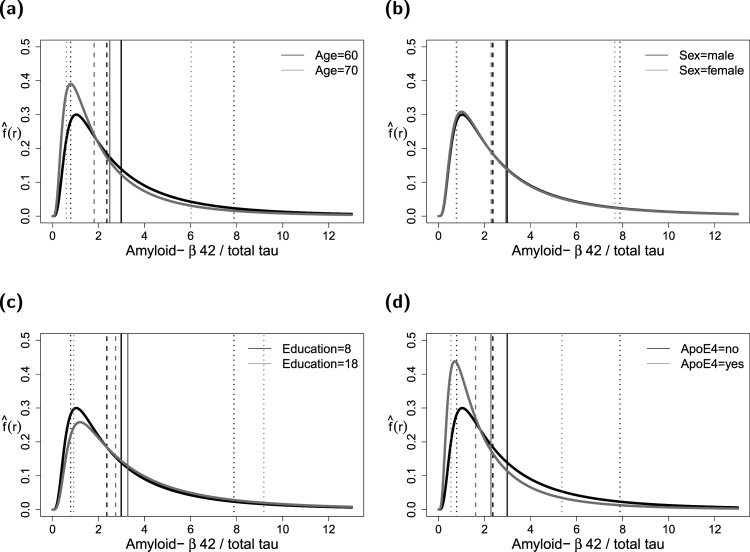
Analysis of the amyloid- β 42/total tau ratios in the DCN study data. The black lines refer to the estimated PDFs for a covariate profile of a randomly selected study participant (60 years of age, Sex = male, Education = 8 years, ApoE ϵ 4 = no). The gray lines refer to a situation where the participant would have been 70 years of age (a), would have been female (b), would have had 18 years of education (c), and would have been a carrier of the ApoE ϵ 4 allele (d). The vertical lines correspond to the estimated mean values (solid), median values (dashed) and 10% and 90% percentiles (dotted). DCN: Dementia Competence Network; PDF: probability density function.

The results obtained from the reduced FCGAM models are shown in Table 5, Figure 6 and Supplemental Figure S6. The upper part of Table 5 refers to the parameter $\Lambda =\lambda_{U}/\lambda_{V}$ , reporting the differences ${\hat{\beta }}_{\Lambda j}:={\hat{\beta }}_{Uj}-{\hat{\beta }}_{Vj}$ . Note that the coefficient estimates are very similar to the respective estimates of the more complex model in Table S1. For example, for amyloid- β 42/total tau one obtains ${\hat{\beta }}_{\Lambda ,\text{ApoE }\epsilon 4}=0.3786$ (Table 5) and ${\hat{\beta }}_{\Lambda ,\text{ApoE}\epsilon 4}=0.2411+0.1406=0.3817$ (Table S1). The credible intervals in Table 5 were obtained by drawing a sample of size 10,000 from the posterior distribution in (14) and by calculating the 2.5% and 97.5% percentiles from the sampled differences $\beta_{Uj}-\beta_{Vj}$ . According to the results of the FCGAM model, there is strong evidence for an effect of the risk factors age and ApoE ϵ 4 on the amyloid- β 42/40 and amyloid- β 42/total tau ratios. As depicted in Figure 6(a) and Supplemental Figure S6(a), both the expected amyloid- β 42/40 ratio and the expected amyloid- β 42/total tau ratio decrease with increasing age, implying a higher risk of progression to AD in older patients. Similarly, the expected ratios of ApoE ϵ 4 carriers are strongly reduced compared to patients not carrying the allele (Figure 6(d) and Supplemental Figures S6(d), confirming the important role of this genetic risk factor in AD progression). The figures also illustrate how the estimated median values as well as the 10% and 90% percentiles of the distributions change with the covariates. In contrast to age and ApoE ϵ 4, Table 5 shows no evidence for an effect of sex and educational level on the two ratio outcomes. These results are in full agreement with the findings by Bergeret al,^12^ who fitted an eGB2 model with amyloid- β 42/40 outcome to the DCN study data.

**Table 5.** Analysis of the amyloid- β 42/40 ratios (left) and the amyloid- β 42/total tau ratios (right) in the DCN study data.

		amyloid- β 42/40		amyloid- β 42/total tau	
Parameter	Covariate	$\hat{\beta }$	95% CI	$\hat{\beta }$	95% CI
Λ	Age	0.0089	[0.0041;0.0137]	0.0256	[0.0156;0.0377]
	Education	−0.0017	[−0.0152;0.0117]	−0.0150	[−0.0461;0.0168]
	Sex (male)	.	.	.	.
	Sex (female)	0.0724	[−0.0073;0.1547]	0.0283	[−0.1544;0.2146]
	ApoE ϵ 4 (no)	.	.	.	.
	ApoE ϵ 4 (yes)	0.1967	[0.1157;0.2766]	0.3786	[0.1965;0.5584]
θ		3.5325	[2.7671;4.2888]	−2.0611	[−2.8064;−1.3073]
$\delta_{U}$		6.0586	[5.1169;7.0218]	5.8090	[4.9062;6.7092]
$\delta_{V}$		10.0151	[8.5182;11.5039]	2.6718	[2.2900;3.0542]

The table presents the coefficient estimates with 95% credible intervals (calculated by the procedure described in Section 2.3), as obtained from fitting FCGAM models with constant association parameter θ . FCGAM: Frank copula with gamma distributed marginal; DCN: Dementia Competence Network.

### 4.6. Comparison of models

**Figure 7.**
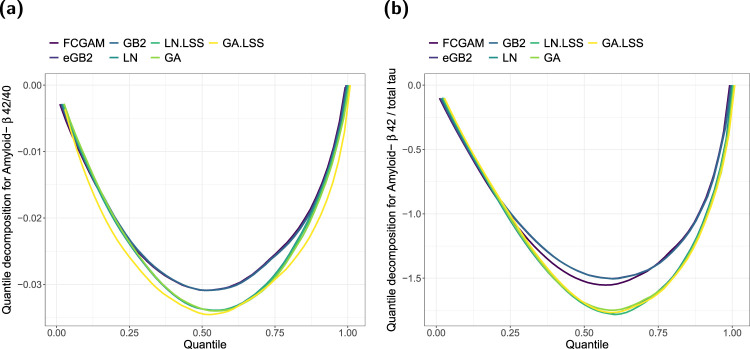
Analysis of the amyloid- β 42/40 ratios (left) and the amyloid- β 42/total tau ratios (right) in the DCN study data. Quantile decomposition of the CRPS for alternative methods based on 1000 samples drawn from the conditional distributions for r∈(0,0.4] (amyloid- β 42/40) and for r∈(0,13] (amyloid- β 42/total tau). The integrated scores were 0.02191 (FCGAM), 0.02191 (eGB2), 0.02192 (GB2), 0.02343 (LN), 0.02343 (LN.LSS), 0.02351 (GA), 0.02493 (GA.LSS) for amyloid- β 42/40 and 1.08400 (FCGAM), 1.06040 (eGB2), 1.06056 (GB2), 1.19883 (LN), 1.20509 (LN.LSS), 1.19365 (GA), 1.19707 (GA.LSS) for amyloid- β 42/total tau. Note: For better readability the curves were jittered to a minimal amount. Higher values indicate better performance. DCN: Dementia Competence Network; FCGAM: Frank copula with gamma distributed marginal; CRPS: continuous ranked probability score.

In a last step of our analysis, we compared the final FCGAM models to the benchmark methods (i) to (vii) already considered in the simulation studies. For all models, we computed the continuous ranked probability score (CRPS) and a corresponding quantile decomposition of the CRPS,^37^ both oriented such that higher values indicate better models. The latter allows to compare the goodness-of-fit with regard to specific quantiles. For this evaluation we drew 1000 samples from the conditional distributions. The results depicted in Figure 7 show that for both ratio outcomes the FCGAM model outperformed the alternatives except for the GB2 and eGB2 models. While for amyloid- β 42/40 the copula-based FCGAM and the models based on the GB2 distribution yielded almost identical scores, for amyloid- β 42/total tau the GB2 and eGB2 model showed a slightly better fit for quantiles <0.7 . Because here the FCGAM model is not advantageous compared to the models designed for positive or zero correlation, this benchmark experiment indicates that the proposed model could still be refined using other marginal distributions or other copulas (see also the discussion in the next section).

## 5. Discussion

The main contribution of this work is a copula-based regression model that serves as a surrogate to relate the ratio of two gamma distributed components to a set of covariates. Conditional copula regression with covariate-dependent copulas is a growing field in the literature see e.g., Emura et al.^38^ and Barone and Dalla Valle^39^ for recent developments. Our model is primarily designed for the analysis of ratio outcomes in medical research, which is an important task, for instance, in neurology,^40^ infectiology^3^ and pharmacology.^41^ Importantly, when biomarker ratios are used as clinical metrics or indicators of clinical outcomes, our model may be used to relate the respective ratio values to a set of risk factors and/or confounding variables. A prototypical example is given by the prognosis of AD progression considering ratios of amyloid- β and total tau protein biomarkers, as presented in Section 4 of this paper.

Conceptually, the FCGAM model developed in this paper has the following advantages: First, by assuming the ratio components to follow univariate gamma distributions, the FCGAM model represents the two biomarkers by real-valued random variables with positive support and right-skewed (marginal) distributions. These distributional characteristics, which are common to most biomarkers encountered in medical research, are directly incorporated in the definition of the proposed copula model. As a consequence, the resulting ratio density incorporates the full information contained in the marginal densities of the components of the ratio. We emphasize that this property does not apply to simpler modeling approaches approximating the ratio by a single log-normal or gamma-distributed variable. In fact, without consideration of the paired components themselves, these approximations inevitably bear the risk of a loss of information (neglected companion).^42^ This issue has been supported by the results of our simulation study (Section 3), which resulted in an increased estimation accuracy of the proposed copula-based approach in all data-generating scenarios. We also stress that linking the two marginal distributions by a copula does in general not restrict our model to the use of two gamma distributions for the ratio components. In fact, although our model can be seen as the most relevant use case in many medical applications, the marginal distributions can in principle be replaced by arbitrary parametric distributions. For instance, our model can in a straightforward manner be extended to situations where one biomarker is discrete or ordinal.

Second, the proposed FCGAM model has a high flexibility regarding the direction of the association between the two ratio components. Importantly, by choosing the Frank copula, the FCGAM model allows for both positive and negative values of the (rank) correlation between the components U and V , thereby improving previous modeling approaches that restricted this correlation to be zero or positive.^12,15^ As demonstrated in the simulation study in Section 3, the FCGAM model indeed performs better in terms of estimation accuracy when the association between U and V is negative. On the other hand, it does not perform worse than the aforementioned approach when the association between U and V is positive.

Third, although the proposed model incorporates the full information contained in the marginal densities $f_{U}$ and $f_{V}$ , it provides a rather simple interpretation of the associations between the ratio U/V and the covariates. This is because the FCGAM model reduces the original five-parameter set $(\lambda_{U},\delta_{U},\lambda_{V},\delta_{V},\theta {)}^{⊤}$ (including all parameters of the marginal densities and the association parameter θ ) to the restricted set $(\Lambda ,\delta_{U},\delta_{V},\theta {)}^{⊤}$ with $\Lambda =\lambda_{U}/\lambda_{V}$ . As a consequence, when treating $\delta_{U}$ , $\delta_{V}$ (and possibly also θ ) as nuisance parameters, the associations between U/V and each of the covariates can be investigated using one-dimensional coefficient estimates and single-parameter hypothesis tests. Similarly, the association between the components U and V has a natural interpretation in terms of Kendall’s rank correlation, being related to θ by the one-to-one relationship given in equation (3).

Beside the flexibility in specifying other marginal distributions than the gamma distribution, the FCGAM model may be extended in many other ways. For example, the Frank copula could be replaced by other copulas noting that the results on ratio densities are also valid for other absolutely continuous copulas; see Ly et al.^33^ Accordingly, a suitable link function for the predictor of θ in (10) needs to be incorporated. When there is particular interest in the tail dependencies of U and V , benchmark experiments to identify the best fitting copula and/or marginal distributions could be performed using resampling techniques (e.g. bootstrapping or subsampling). It should be noted, however, that other copulas from the literature might be less flexible regarding the range of θ and thus also the range of possible associations between the components U and V ; see e.g. Ghosh, Bhuyan and Finkelstein^43^ for a recent overview of copulas allowing for modeling negative dependence. For example, it is not possible to model negative associations between U and V using non-rotated Gumbel or Joe copulas.

## Supplemental Material

sj-pdf-1-smm-10.1177_09622802241313293 - Supplemental material for Modeling the ratio of correlated biomarkers using copula regression Supplemental material, sj-pdf-1-smm-10.1177_09622802241313293 for Modeling the ratio of correlated biomarkers using copula regression by Moritz Berger, Nadja Klein, MichaelWagner and Matthias Schmid in Statistical Methods in Medical Research
