# Supplementary material for: Modeling the ratio of correlated biomarkers using copula regression
Source: Stat Methods Med Res. 2025 Feb 11;34(5):968–85. doi: 10.1177/09622802241313293 (PMC12177203; doi:10.1177/09622802241313293)
Supplement: sj-pdf-1-smm-10.1177_09622802241313293 - Supplemental material for Modeling the ratio of correlated biomarkers using copula regression [file sj-pdf-1-smm-10.1177_09622802241313293.pdf]

Journal Title
XX(X):1–8
©The Author(s) 2024
Reprints and permission:
sagepub.co.uk/journalsPermissions.nav
DOI: 10.1177/ToBeAssigned
www.sagepub.com/
SAGE

Supplement to

Modeling the Ratio of Correlated Biomarkers Using Copula Regression

Moritz Berger[1], Nadja Klein[2], Michael Wagner[3] and Matthias Schmid[1]

[1]Department of Medical Biometry, Informatics and Epidemiology, Faculty of Medicine, University of Bonn
[2]Scientific Computing Center, Karlsruhe Institute of Technology
[3]German Center for Neurodegenerative Diseases

Corresponding author:
Scientific Computing Center, Karlsruhe Institute of Technology, Zirkel 2, 76131 Karlsruhe, Germany
Email: nadja.klein@kit.edu

A Proof of Proposition 2

By Proposition 1, the PDF of the ratio $R = U/V$ is given by

$$
\begin{aligned}
f_R(r;\lambda_U,\lambda_V,\delta_U,\delta_V,\theta) &= \int_0^1 c_\theta \left[F_U(r\,F_V^{-1}(s;\lambda_V,\delta_V);\lambda_U,\delta_U), s\right] \\
&\quad \times \left|F_V^{-1}(s;\lambda_V,\delta_V)\right| \, f_U(rF_V^{-1}(s;\lambda_V,\delta_V),\lambda_U,\delta_U)\, ds \\
&= \int_0^1 c_\theta \left\{ \frac{1}{\Gamma(\delta_U)} \gamma\left(\delta_U, \lambda_U\, r\, F_V^{-1}(s;\lambda_V,\delta_V)\right), s \right\} \\
&\quad \times F_V^{-1}(s;\lambda_V,\delta_V)\, \frac{\lambda_U^{\delta_U}}{\Gamma(\delta_U)} \left(r\,F_V^{-1}(s;\lambda_V,\delta_V)\right)^{\delta_U - 1} \\
&\quad \times \exp\left(-\lambda_U\, r\, F_V^{-1}(s;\lambda_V,\delta_V)\right)\, ds \\
&= \int_0^1 c_\theta \left\{ \frac{1}{\Gamma(\delta_U)} \gamma\left(\delta_U, \lambda_U\, r\, \frac{\gamma^{-1}\left(\delta_V, \Gamma(\delta_V)\, s\right)}{\lambda_V}\right), s \right\} \\
&\quad \times \frac{\lambda_U^{\delta_U}}{\Gamma(\delta_U)} \left(\frac{\gamma^{-1}\left(\delta_V, \Gamma(\delta_V)\, s\right)}{\lambda_V}\right)^{\delta_U} r^{\delta_U - 1} \\
&\quad \times \exp\left(-\lambda_U\, r\, \frac{\gamma^{-1}\left(\delta_V, \Gamma(\delta_V)\, s\right)}{\lambda_V}\right) ds \\
&\overset{\Lambda = \lambda_U/\lambda_V}{=} \int_0^1 c_\theta \left\{ \frac{1}{\Gamma(\delta_U)} \gamma\left(\delta_U, r\,\Lambda\,\gamma^{-1}\left(\delta_V, \Gamma(\delta_V) s\right)\right), s \right\} \\
&\quad \times \frac{\Lambda^{\delta_U}\, r^{(\delta_U - 1)}}{\Gamma(\delta_U)} \left[\gamma^{-1}\left(\delta_V, \Gamma(\delta_V) s\right)\right]^{\delta_U} \\
&\quad \times \exp\left[-r\,\Lambda\,\gamma^{-1}\left(\delta_V, \Gamma(\delta_V) s\right)\right]\, ds \\
&= f_R(r;\Lambda,\delta_U,\delta_V,\theta)\,,
\end{aligned}
$$

where $\gamma(\cdot,\cdot)$ denotes the lower incomplete gamma function. □

B Further Simulation Results

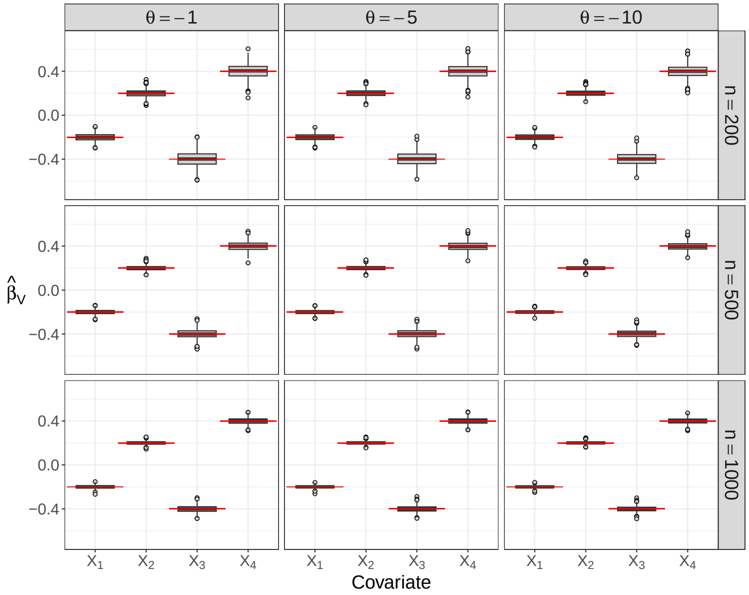

Figure S1. Point estimates of the FCGAM coefficients in *Simulation Study 1*. The boxplots visualize the MLEs of the coefficients $\beta_{V1} = -0.2$, $\beta_{V2} = 0.2$, $\beta_{V3} = -0.4$ and $\beta_{V4} = 0.4$ that were obtained from fitting the FCGAM model to 1000 data sets of size n each. The red lines refer to the true values of the coefficients.

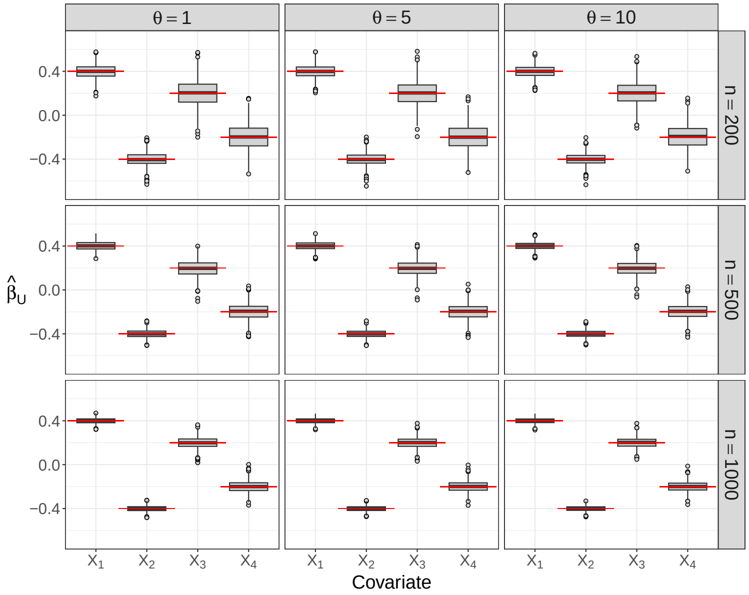

Figure S2. Point estimates of the FCGAM coefficients in *Simulation Study 2*. The boxplots visualize the MLEs of the coefficients $\beta_{U1} = 0.4$, $\beta_{U2} = -0.4$, $\beta_{U3} = 0.2$ and $\beta_{U4} = -0.2$ that were obtained from fitting the FCGAM model to 1000 data sets of size n each. The red lines refer to the true values of the coefficients.

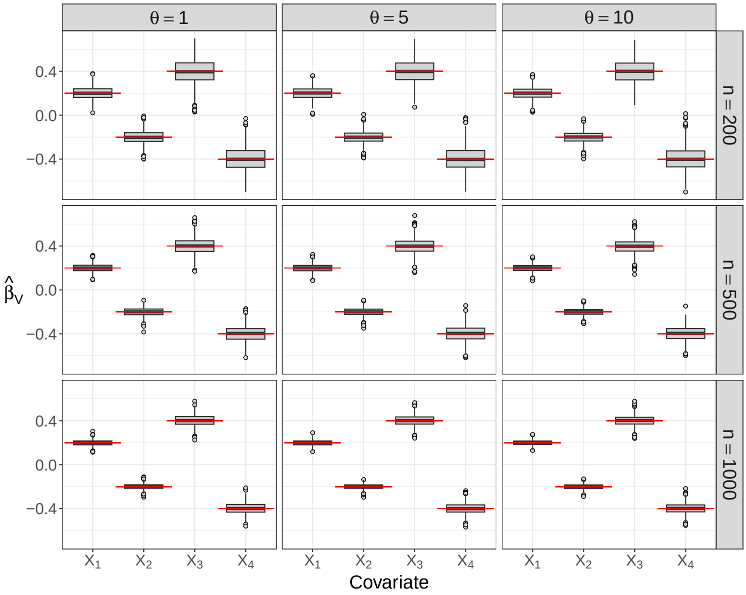

Figure S3. Point estimates of the FCGAM coefficients in *Simulation Study 2*. The boxplots visualize the MLEs of the coefficients $\beta_{V1} = 0.2$, $\beta_{V2} = -0.2$, $\beta_{V3} = 0.4$ and $\beta_{V4} = -0.4$ that were obtained from fitting the FCGAM model to 1000 data sets of size n each. The red lines refer to the true values of the coefficients.

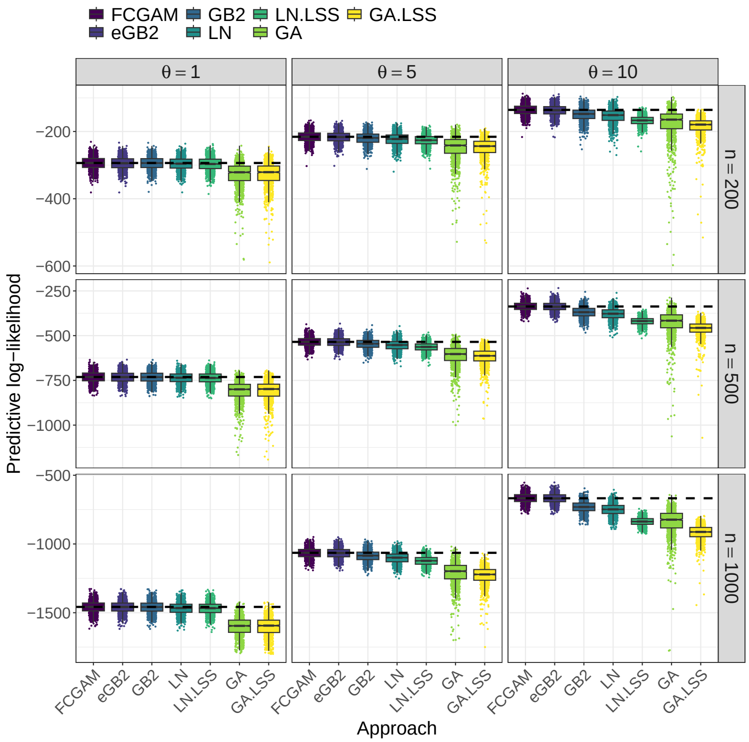

Figure S4. Comparison of the FCGAM model to alternative methods in *Simulation Study 2*. The boxplots visualize the predictive log-likelihood values obtained from the FCGAM model and from the benchmark methods (ii) to (vii). All models were fitted to 1000 independent data sets and evaluated on independently generated test data sets of the same size. In each panel, the dashed horizontal line indicates the median predictive log-likelihood of the best performing method.

C Further Results of the Analysis of the DCN Study Data

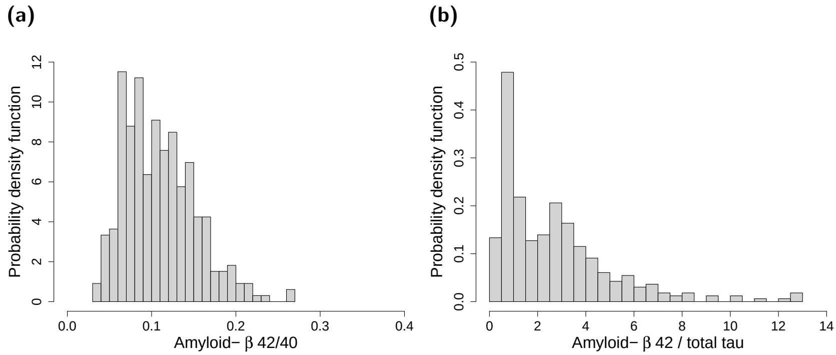

Figure S5. Analysis of the DCN study data. Distribution of the amyloid-β 42/40 ratios (a) and the amyloid-β 42/total tau ratios (b) in patients with MCI ($n = 330$).

Table S1. Analysis of the amyloid-β 42/40 ratios (left) and the amyloid-β 42/total tau ratios (right) in the DCN study data. The table presents the coefficient estimates with 95% credible intervals (calculated by the procedure described in Section 2.3), as obtained from fitting FCGAM models with covariate-dependent association parameter θ.

		**amyloid-β 42/40**		**amyloid-β 42/total tau**	
Parameter	Covariate	$\hat{\beta}$	95% CI	$\hat{\beta}$	95% CI
λ_U	Age	0.0073	[0.0019; 0.0127]	0.0092	[0.0037; 0.0148]
	Education	0.0079	[−0.0071; 0.0224]	0.0035	[−0.0123; 0.0192]
	Sex (male)	.	.	.	.
	Sex (female)	0.0017	[−0.0905; 0.0933]	−0.0247	[−0.1178; 0.0680]
	ApoE ϵ4 (no)	.	.	.	.
	ApoE ϵ4 (yes)	0.1774	[0.0871; 0.2681]	0.2411	[0.1503; 0.3325]
λ_V	Age	−0.0015	[−0.0056; 0.0025]	−0.0160	[−0.0242; −0.0079]
	Education	0.0100	[−0.0013; 0.0212]	0.0166	[−0.0071; 0.0406]
	Sex (male)	.	.	.	.
	Sex (female)	−0.0751	[−0.1460; −0.0061]	−0.0746	[−0.2127; 0.0663]
	ApoE ϵ4 (no)	.	.	.	.
	ApoE ϵ4 (yes)	−0.0141	[−0.0811; 0.0526]	−0.1406	[−0.2745; −0.0065]
θ	Age	0.0369	[−0.0645; 0.1347]	0.0637	[−0.0356; 0.1640]
	Education	0.1247	[−0.1453; 0.3928]	0.0165	[−0.2416; 0.2750]
	Sex (male)	.	.	.	.
	Sex (female)	−0.5679	[−2.1432; 1.0406]	−0.9324	[−2.4944; 0.6044]
	ApoE ϵ4 (no)	.	.	.	.
	ApoE ϵ4 (yes)	0.5610	[−0.9635; 2.0978]	−1.2897	[−2.8033; 0.1928]
δ_U		5.7822	[4.9088; 5.6759]	5.6900	[4.8084; 6.5605]
δ_V		10.0740	[8.4946; 11.6237]	2.6023	[2.2232; 2.9831]

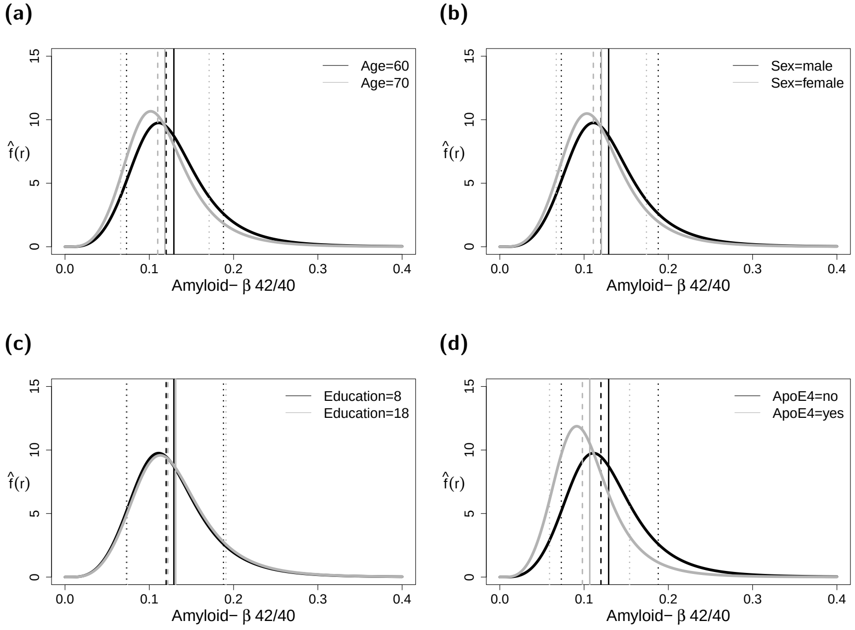

Figure S6. Analysis of the amyloid-β 42/40 ratios in the DCN study data. The black lines refer to the estimated PDFs for a covariate profile of a randomly selected study participant (60 years of age, Sex = male, Education = 8 years, ApoE $\epsilon 4$ = no). The gray lines refer to a situation where the participant would have been 70 years of age (a), would have been female (b), would have had 18 years of education (c), and would have been a carrier of the ApoE $\epsilon 4$ allele (d). The vertical lines correspond to the estimated mean values (solid), median values (dashed) and 10% and 90% percentiles (dotted).
